# Changes in Office Blood Pressure Control, Augmentation Index, and Liver Steatosis in Kidney Transplant Patients after Successful Hepatitis C Infection Treatment with Direct Antiviral Agents

**DOI:** 10.3390/jcm9040948

**Published:** 2020-03-30

**Authors:** Aureliusz Kolonko, Joanna Musialik, Jerzy Chudek, Magdalena Bartmańska, Natalia Słabiak-Błaż, Agata Kujawa-Szewieczek, Piotr Kuczera, Katarzyna Kwiecień-Furmańczuk, Andrzej Więcek

**Affiliations:** 1 Department of Nephrology, Transplantation and Internal Medicine, Medical University of Silesia, Francuska 20/24, 40-027 Katowice, Poland; jmusialik@sum.edu.pl (J.M.); bartmanska.m@gmail.com (M.B.); nataliablaz@gazeta.pl (N.S.-B.); agata.szewieczek@gmail.com (A.K.-S.); p.m.kuczera@gmail.com (P.K.);; 2 Department of Internal Medicine and Oncological Chemotherapy, Medical University of Silesia, Reymonta 8, 40-035 Katowice, Poland; chj@poczta.fm

**Keywords:** blood pressure, eradication, interferon-free regimen, hepatitis C infection, kidney transplant

## Abstract

Hepatitis C virus (HCV) infection in kidney transplant recipients (KTRs) can be successfully treated with direct antiviral agents (DAA). The aim of our study was to analyze different measures of vascular function during and after the DAA treatment. As we have observed the improvement of blood pressure (BP) control in some individuals, we have conducted an analysis of potential explanatory mechanisms behind this finding. Twenty-eight adult KTRs were prospectively evaluated before and 15 months after start of DAA therapy. Attended office BP (OBP), augmentation index (AIx), pulse wave velocity (PWV), flow-mediated dilation (FMD), liver stiffness measurement (LSM), and liver steatosis assessment (controlled attenuation parameter (CAP)) were measured. In half of the patients, improvement of OBP control (decline of systolic BP by at least 20 mmHg or reduction of the number of antihypertensive drugs used) and parallel central aortic pressure parameters, including AIx, was observed. There was a significant decrease in CAP mean values (241 ± 54 vs. 209 ± 30 dB/m, *p* < 0.05) only in patients with OBP control improvement. Half of our KTRs cohort after successful HCV eradication noted clinically important improvement of both OBP control and central aortic pressure parameters, including AIx. The concomitant decrease of liver steatosis was observed only in the subgroup of patients with improvement of blood pressure control.

## 1. Introduction

Arterial hypertension is highly prevalent in kidney transplant recipients (KTRs) as a consequence of common pretransplant hypertension and as an additional effect of immunosuppressive medications [1,2]. It has been shown that blood pressure (BP) control is suboptimal (systolic BP > 140 mmHg) in 50% of KTRs [3,4,5]. In addition, higher BP values were associated with reduced graft and patient survival [5]. Notably, cardiovascular disease is the primary cause of mortality among kidney transplant recipients, mostly due to long-term consequences of chronic kidney disease (CKD) [6]. CKD-related systemic inflammation, calcium-phosphate abnormalities, and oxidative stress promote endothelial dysfunction, vascular calcification, and accelerated atherosclerosis in addition to the traditional risk factors [7,8]. Vascular injury caused by the uremic milieu results in an increased arterial stiffness and reduced flow-mediated dilation (FMD) [9,10]. 

Chronic hepatitis C virus (HCV) infection has been shown to independently worsen posttransplant survival [11]. HCV infection is a risk factor for increased aortic stiffness and cardiovascular events in dialysis patients [12], whereas advanced HCV-derived liver fibrosis is associated with increased endothelial dysfunction, independently of common cardiovascular risk factors [13]. On the other hand, patients with chronic HCV infection also demonstrate impaired autonomic nervous system function [14]. All the above disturbances caused by coexisting HCV infection may partially worsen blood pressure control in KTRs. 

A few years ago a breakthrough in the treatment of chronic hepatitis C occurred. The previous interferon-based therapy, which was contraindicated for kidney transplanted patients due to increased risk of organ rejection, was replaced by new, direct acting antiviral (DAA) drug regimens. In our observation, the effectiveness of this therapy, based on sofosbuvir, reached 100% [15]. We hypothesized that the successful eradication of HCV infection may directly or indirectly improve the endothelial function. In the present study, patients were prospectively evaluated regarding different measures of their vascular function, including endothelial function, arterial stiffness measurement, and blood pressure control. Concurrently, we assessed the liver stiffness and steatosis before and after the DAA treatment. As we observed the improvement of blood pressure control in some individuals, we conducted an analysis of potential explanatory mechanisms behind this finding. 

## 2. Material and Methods

### 2.1. Study Group

The study protocol was accepted by the Bioethics Committee of the Medical University of Silesia in Katowice (KNW/0022/KB1/119/16) and all participants provided written informed consent. The study was conducted in accordance with the Declaration of Helsinki. We prospectively studied all eligible adult kidney transplant recipients (KTRs) who completed treatment with DAA therapy due to HCV infection and completed both baseline and follow-up examination at least 12 months after the start of DAA therapy. 

### 2.2. Clinical, Anthropometric, and Laboratory Measurements

Body weight and height were measured following standard procedures and body mass index (BMI) was then calculated (kg/m^2^). Body surface area (BSA) was calculated according to the DuBois formula and was expressed in m^2^.

Kidney graft function was measured by the estimated glomerular filtration rate (eGFR), which was calculated according to the abbreviated Modification of Diet in Renal Disease formula. 

HOMA-IR (Homeostatic model assessment of insulin resistance) was calculated to assess insulin resistance. 

HCV RNA was measured with COBAS^®^ AmpliScreen HCV v.1.0, with lower limit of detection of 15 IU/mL (Roche Diagnostics, USA). HCV genotyping and viral load was performed with Linear Array Genotyping Test and COBAS^®^TaqMan^®^ Quantitative test v.1.0, with lower limit of detection of 21 IU/mL (TaqMan; Roche Diagnostics, Branchburg, NJ, USA).

Concentrations of blood glycated hemoglobin (HbA_1C_), serum creatinine, total cholesterol, triglycerides, and total bilirubin concentrations, as well as aspartate aminotransferase (AST), alanine aminotransferase (ALT), and gamma glutamyl transpeptidase (GGT) activity were routinely measured during standard outpatient visits. Additional blood samples were withdrawn in a closed system into tubes containing citrate and ethylenediaminetetraacetic acid (EDTA) for nonroutine analyses. The tubes were allowed to stand for 2 h at room temperature, then centrifuged (15 min, 3000 rpm), and finally plasma aliquots were preserved at −70 °C. 

The plasma concentrations of high-sensitivity C-reactive protein (hsCRP) were assessed with the use of an enzyme-linked immunosorbent assay (ELISA) (Immundiagnostic AG, Bensheim, Germany), with the limit of quantification (LoQ) of 0.09 mg/L, intra-assay variation <6%, and inter-assay variation <11.6%. Plasma concentrations of interleukin-6 (IL-6) were assessed with an ELISA (R&D Systems, Minneapolis, MN, USA) with a LoQ of 0.7 pg/mL, intra-assay variation <4.2%, and inter-assay variation <6.4%. Plasma concentrations of fibroblast growth factor 21 (FGF-21) were assessed with an ELISA (Biovendor, Brno, Czech Republic) with a LoQ of 7 pg/mL, intra-assay variation <2.0%, and inter-assay variation <3.3%. Plasma concentrations of C-peptide and insulin were measured using a Cobas E411 analyzer with intermediate precision <5.0% and <2.8%, respectively. 

Each study examination consisted of the measurement of office BP and central arterial pressure parameters. Echocardiography, pulse wave velocity (PWV), FMD, liver stiffness measurement (LSM), and liver steatosis assessment (controlled attenuation parameter (CAP)) were also performed at the same time points. 

Attended office blood pressure measurements (OBP) were performed three times in the sitting position after more than a 5-min rest in the sitting position on the arm without arterio-venous fistula at the beginning of the study. Patients with systolic BP values ≥140 mmHg and/or diastolic BP values ≥90 mmHg or those who received antihypertensive medication were diagnosed as hypertensive. For the present post hoc analysis, the OBP control improvement was defined as the decline of attended office systolic pressure by at least 20 mmHg without pharmacotherapeutic changes or the reduction of antihypertensive treatment due to the BP decline during the follow-up period. The cut-off value (20 mmHg) was established based on doubled maximum measurement error of 10 mmHg [16,17]. Patients were divided into two study subgroups based on the BP control improvement during the follow-up period. 

### 2.3. Echocardiography

Echocardiographic measurements were performed using Toshiba Xario 100 Diagnostic Ultrasound System (Toshiba, Toshiba Medical System Corporation, Tochigi 324-8550, Japan). M-mode and two-dimensional measurements were performed as recommended by the American Society of Echocardiography [18], including left ventricular end-diastolic and end-systolic diameters, intraventricular septum, and posterior wall end-diastolic thickness. Left ventricular mass (LVM) was calculated according to the Devereux formula [19]. LVM was indexed for BSA (LVMI).

### 2.4. Brachial Artery Flow-Mediated Dilation

FMD was measured in the morning, after 10 min of lying in a quiet dimmed room using the Toshiba Xario 100. During the examination, the patients rested in a seated position with their forearms and backs supported. A manual sphygmomanometer cuff was placed on the arm without arteriovenous fistula and the diameter (lumen) of the brachial artery was measured with a linear transducer. The cuff was then inflated at approximately 50 mmHg above the current systolic pressure for 5 min [20]. After the deflation of the cuff, the serial measurements during diastole were recorded and the widest dilation of the brachial artery was used for FMD calculation: FMD% = (A − B)/B × 100%, where A is the diameter of the artery during reactive hyperemia, and B is the initial diameter of the artery.

Non-endothelial dependent vasodilation (NMD) was assessed after at least a 15-min rest from the FMD acquisition. Similarly to FMD measurements, vessel diameters were assessed before and after sublingual nitroglycerin (400 μg) application (Nitromint (glyceroli trinitras), Proterapia, Poland). Of importance, as we observed unexpected reduction of NMD values in some patients, we repeated all NMD measurements using a double dose of nitroglycerin, i.e., 800 μg, after approximately 6 months from the previous follow-up series. 

### 2.5. Central Aortic Pressure and Arterial Stiffness Measurement

Pulse waveform analysis was performed with the commercially available SphygmoCor 2000 (AtCor Medical, Sydney, Australia). Peripheral pressure waveforms were recorded from the radial artery using applanation tonometry. After the acquisition of at least 20 sequential waveforms, a validated generalized transfer function was used to generate the corresponding central aortic pressure waveform. Central blood pressure measurements, including central aortic systolic and diastolic blood pressure, central pulse pressure, and augmentation index were performed. Augmentation index was then normalized according to the heart rate (AIx@75). Only high-quality recordings, defined as an in-device quality index greater than 80% and visually acceptable curves by the investigator, were included in the analysis. The entire pulse wave analysis was performed in the sitting position under standardized conditions in the morning hours, after at least 15 min of rest in the supine position.

Arterial stiffness was also assessed using SphygmoCor 2000 placed over the carotid and femoral arteries. Pressure signals were calibrated using brachial BP and PWV was calculated as the time of the pulse wave between the diagnosed points (distance (m)/time (s)). 

### 2.6. Liver Elastography

The controlled attenuation parameter (CAP) and liver stiffness measurement (LSM) were performed using transient elastography with a M-probe (FibroScan 502 Touch, Echosense, Paris, France). The operator was a technician certified by Echosense and unaware of patient status. The measurements were performed using a 3.5-MHz standard probe on the right hepatic lobe through the intercostal spaces with the patient lying in a supine position. As recommended by the manufacturer, 10 successful measurements were performed for each patient and only those with a success rate of at least 60% and an interquartile range/median value of less than 0.3 were considered reliable. The final CAP and liver stiffness were expressed in dB/m and kPa, respectively [21,22]. 

### 2.7. Data and Statistical Analysis 

Statistical analyses were performed using the STATISTICA 13.0 PL for Windows software package (StatSoft Poland, Cracow, Poland). The values were presented as mean values with 95% confidence interval (CI) (for variables with normal distribution), medians with Q25-Q75 quartile values (for variables with not normal distribution), or frequencies. Comparisons between groups were done by using the Student t-test for quantitative variables or the χ^2^ test for qualitative variables. Variables with not normal distribution were compared using the Mann–Whitney U test. The comparison of baseline and follow-up values was performed using the Student t-test, or the Wilcoxon test for variables with not normal distribution. Correlation coefficients were calculated using the Pearson test. Due to its not normal distribution, FGF-21 data were logarithmized before the correlation analyses. In all statistical tests, ‘*p*’ values below 0.05 were considered as statistically significant. 

## 3. Results

### 3.1. Study Group

**Figure 1:**
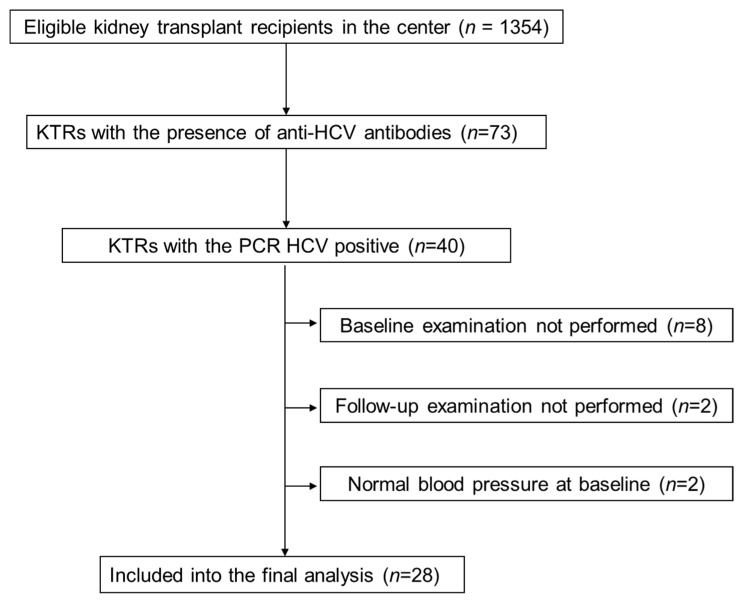
Study flow chart. KTRs, kidney transplant recipients; HCV, hepatitis C virus; PCR, polymerase chain reaction.

Out of all 73 KTRs with the presence of anti-HCV antibodies, HCV-RNA was detected in 40 patients. These patients were qualified to further HCV genotyping and viral load. Out of them, 8 patients had started DAA therapy without baseline examination planned for the present study and were not included in the final analysis. The other 32 patients were treated with the DAA anti-HCV protocol, including 8 with a 6-month regimen and 24 patients with a 3-month regimen based on sofosbuvir. The DAA regimen was shortened in the later enrolled patients as the treatment recommendations were updated during the study period. Out of this group, 4 KTRs were excluded: 2 of them were normotensive prior to HCV treatment and 2 others did not finalize the study protocol (Figure 1). Hence, the final study group consisted of 28 hypertensive patients who completed DAA therapy and both baseline and follow-up examinations. In all study patients, the diagnosis of HCV infection was established prior to kidney transplantation. In all patients, HCV viremia was not detectable after the first month of treatment and they all reached sustained virologic response at 48 weeks from DAA treatment start (SVR48) time point. The clinical characteristics of the study patients are presented in Table 1. 

### 3.2. Study Subgroups Based on Blood Pressure Control

In the follow-up period, half of the patients showed an improvement of OBP control (subgroup 1). Patients with OBP improvement had initially higher systolic BP (SBP) (*p* = 0.02), but similar diastolic BP (DBP) (Table 2). In addition, they received more antihypertensive medications (mean: 2.5 vs. 1.9 drugs) before the start of DAA therapy; however, this difference was not statistically significant. The observed overall SBP (Δ −20.4, 95% CI, −26.2 to −14.6 mmHg) and DBP (Δ −12.5, 95% CI, −16.5 to −8.5 mmHg) decline in subgroup 1 was obtained despite the reduction in the number of antihypertensive drugs in 9 subjects. We also observed mild reduction in SBP (Δ −5.2, 95 % CI, −9.7 to −0.8 mmHg) and DBP (Δ −4.6, 95% CI, −9.6 to 0.3 mmHg) in the second subgroup.

At baseline, the antihypertensive treatment was used in all patients: Beta-blockers in 67.9%, calcium channel blockers in 25%, angiotensin-converting enzyme inhibitor or angiotensin receptor blocker in 32.1%, alpha-blocker in 25%, and diuretics in 32.1% of study participants. The structure of antihypertensive medication classes was similar in both study subgroups (data not shown).

Both study subgroups did not differ in respect to age, gender, BMI, pretransplant dialysis vintage, time after kidney transplantation, and the occurrence of diabetes (Table 1). The time from diagnosis of HCV infection to DAA treatment was significantly longer in the subgroup 2 (Table 1), however, the percentage of patients with HCV infection lasting more than 13.7 years (mean value in the whole group) did not differ between study subgroups (*p* = 0.13). Out of whole group, only 4 patients were previously treated with interferon-based anti-HCV regimens. There were no differences in regards to baseline values of serum lipid concentrations, fasting glucose and insulin concentrations, glycated hemoglobin, and HOMA-IR values between subgroups (Supplementary Table S1).

Also, the HCV genotypes were similar in both subgroups, including 11 patients with genotype 1b in each group. The percentage of patients with advanced fibrosis, defined based on a METAVIR score >2, was comparable (28.6 vs. 38.5%, *p* = 0.59). The mean time between baseline and follow-up study examinations was also similar (15.0 ± 1.4 vs. 15.9 ± 2.1 months, *p* = 0.22). 

Both study subgroups were similar in respect to calcineurin inhibitor (CNI) structure (Table 1). There were no CNI-type conversions during the whole study period. At baseline, median cyclosporine (CyA) doses were comparable (100 (100–125) mg in subgroup 1 vs. 125 (100–150) mg in subgroup 2, *p* = 0.60), whereas median tacrolimus (Tc) doses were significantly greater in subgroup 2 (4.0 (2.5–6.0) mg vs. 1.0 (1.0–2.0) mg in subgroup 1, *p* < 0.05). Notably, median CyA (97 (70–128) vs. 125 (100–146) ng/mL, respectively; *p* = 0.30) and Tc (7.1 (6.1–7.4) vs. 8.4 (6.7–8.7) ng/mL, respectively; *p* = 0.22) blood trough concentrations were similar. During and after DAA treatment, the improved liver function resulted in the reduction of calcineurin inhibitor (cyclosporine or tacrolimus) blood trough concentrations as compared with baseline values, which were similar in both study subgroups (−21.4 (−37.2 to −5.7) vs. −11.3 (−33.5 to 10.9)%, respectively; *p* = 0.42) and required individual dose adjustments in 61% of patients (*n* = 17) as soon as after one month of therapy. The consecutive CNI dose adjustments were made at physician discretion and were guided by the drug blood concentration, to prevent the CyA level decreasing below 70 ng/mL or Tc level decreasing below 5 ng/mL. Overall, the median CNI dose changes in both study subgroups were similar (13.4 (interquartile range (IQR) 0–25) vs. 25 (0–75)%, respectively; *p* = 0.26). Also, the absolute median dose changes of CyA (0 (0–75) vs. 25 (12.5–25) mg, respectively; *p* = 0.73) and Tc (0.5 (0.5–1.0) vs. 0 (−1.5–0.5) mg, respectively; *p* = 0.26) were similar. 

### 3.3. Liver Function Tests and Liver Morphologic Assessments

**Figure 2:**
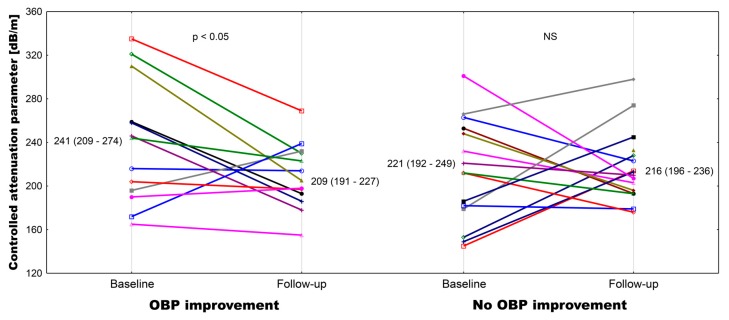
Individual plot of the controlled attenuation parameter at baseline and after the follow-up in patients with and without attended office blood pressure control improvement.

At baseline, there was a numerical difference in HCV viremia between subgroups (Table 1), but neither HCV viremia nor baseline liver function tests differed significantly. In both subgroups, there was a significant reduction in aminotransferases and GGT activities after DAA treatment (Supplemental Materials Table S2). Liver elastography measurements were performed with a success rate near 100% (only 3 out of 28 patients needed 11 total measurements to obtain 10 valid results). Baseline liver stiffness measurements did not differ between subgroups and remained unchanged thereafter. On the contrary, CAP values only declined significantly in subgroup 1 (Supplementary Materials Table S2 and Figure 2).

### 3.4. Central Blood Pressure Parameters

Both baseline and follow-up values of central aortic systolic pressure and central aortic pulse pressure correlated with corresponding values of OBP (baseline *r* = 0.669, *p* < 0.001, follow-up *r* = 0.557, *p* < 0.01 for SBP; and baseline *r* = 0.730, *p* < 0.001, follow-up *r* = 0.502, *p* < 0.01 for pulse pressure, respectively). There were also significant correlations between baseline augmentation index values standardized to heart rate (AIx@75) and both baseline and follow-up values of office SBP and pulse pressure (baseline *r* = 0.660, *p* < 0.001 and *r* = 0.562, *p* < 0.01, respectively; follow-up *r* = 0.484, *p* < 0.01 and *r* = 0.545, *p* < 0.01, respectively) (Table 3). 

Parallel to OBP, we observed a 15.4% decline in aortic systolic pressure, a 12.1% decline in aortic diastolic pressure, and a 21.7% decline in aortic pulse pressure in subgroup 1 after successful DAA treatment (Table 3). Of note, AIx@75 values decreased significantly only in subgroup 1. On the contrary, only a mild decline of systolic aortic pressure (7.2%) was observed in subgroup 2. There was a positive correlation between the percentage change in CAP values and both the change in central aortic systolic pressure (*r* = 0.438, *p* < 0.05) and the change in AIx@75 (*r* = 0.446, *p* < 0.05). Of note, both baseline (*r* = 0.479, *p* < 0.05) and follow-up (*r* = 0.431, *p* < 0.05) CAP results correlated with corresponding BMI values.

### 3.5. Arterial Structural and Functional Measurements

At baseline and in the follow-up period, PWV values were similar in both subgroups (Table 3). FMD values were stable in both subgroups, while NMD measured using the standard (400 μg) dose of nitroglycerin did not change in subgroup 1, but showed a significant reduction in subgroup 2 (Table 3). However, in an additional repeated NMD measurement with a double dose of nitroglycerin there was no significant NMD change compared with baseline values in both study subgroups.

### 3.6. Cardiac Parameters

At baseline, both mean LVM (210 (169–250) vs. 211 (173–250) g, *p* = 0.84)) and mean LVMI (113 (94–131) vs. 114 (95–133) g/m^2^, *p* = 0.98) were comparable in subgroups 1 and 2, respectively. Similarly, there were no differences in the follow-up LVM and LVMI values (0.70 and 0.87, respectively), and their absolute changes during the study period did not differ significantly (LVM 7.9 (−23.5 to 39.2) vs. −0.3 (−44.4 to 43.8), *p* = 0.57; LVMI 3.3 (−13.4 to 19.9) vs. −1.3 (−23.2 to 20.6) g/m^2^, *p* = 0.70).

### 3.7. Inflammatory Markers

The baseline median of CRP concentration was slightly higher in subgroup 1 (1.7 (IQR 1.1–2.4) vs. 0.9 (0.4–1.4) mg/L in subgroup 2, with borderline significance (*p* = 0.07)). At follow-up assessment, there was a borderline increase of median hsCRP in subgroup 2 (3.6 (0.5–6.7), *p* = 0.06), whereas no difference was noted in subgroup 1 (1.8 (1.5–3.0), *p* = 0.33). There were no significant differences in IL-6 concentration median values, both at baseline (2.7 (2.2–3.2) vs. 2.2 (1.8–2.5) pg/mL, *p* = 0.23) and at the follow-up examination (2.7 (1.7–4.1) vs. 2.6 (1.7–5.7) pg/mL, *p* = 0.76). Levels did not change significantly during the study period.

### 3.8. Fibroblast Growth Factor 21 Levels

Median values of FGF-21 measured at follow-up increased significantly in subgroup 1 (411 (204–706) vs. 215 (120–535) pg/mL at baseline, *p* < 0.01), while they remained unchanged in subgroup 2 (189 (147–481) vs. 226 (116–267) pg/mL at baseline, *p* = 0.22). The baseline number of antihypertensive drugs was borderline associated with log values of baseline FGF-21 (*r* = 0.375, *p* = 0.05) and significantly associated with follow-up values of FGF-21 (*r* = 0.467, *p* < 0.05). However, the log values of both baseline and follow-up FGF-21 did not correlate with baseline SBP or DBP values, whereas they correlated significantly with baseline central systolic aortic pressure (*r* = 0.388, *p* < 0.05, and *r* = 0.434, *p* < 0.05, for baseline and follow-up FGF-21 log values, respectively) and borderline with central diastolic aortic pressure (*r* = 0.357, *p* < 0.05, and *r* = 0.385, *p* < 0.05, respectively). Moreover, the log values of follow-up FGF-21 negatively correlated with the change of central systolic and diastolic aortic pressures (*r* = −0.361 and *r* = −0.367, respectively) with both *p* values = 0.06.

## 4. Discussion

In the present study, we demonstrated the clinically important improvement of blood pressure control, defined as the decline of attended office systolic pressure by at least 20 mmHg without pharmacotherapeutic changes or the reduction of antihypertensive treatment due to the BP decline, in half of our cohort of HCV-infected stable kidney transplant recipients after successful HCV eradication with sofosbuvir-based DAA therapy. In this subgroup of patients, we observed the reduction of systolic BP values despite the de-escalation of antihypertensive treatment in 9 out of 14 subjects. Moreover, we also documented the parallel decline of central aortic pressure parameters, obtained with the Sphygmo-Cor device, including augmentation index, in patients with improved BP control. Finally, we noted the corresponding decrease of liver steatosis, measured by the controlled attenuation parameter, in this subgroup of patients, which may suggest the potential underlying mechanism of beneficial vascular changes. 

We are aware that nowadays a majority of dialysis patients with previously diagnosed HCV infection have already been successfully treated with DAA regimen even before they get a kidney transplant. Nevertheless, the confirmation of relatively moderate BP control improvement in patients being currently on dialysis would be much more difficult due to procedural-related fluctuations in volemia. Thus, the present study findings may help to elucidate or confirm some mechanisms linking the HCV infection with BP control in CKD patients. 

There are several possible pathomechanisms linking the chronic HCV infection with elevated blood pressure and increased cardiovascular risk, including increased arterial stiffness [23], endothelial dysfunction [13], and the direct effect of liver steatosis on BP regulation [24]. The virus plays an etiological and pathogenic role in the development of vasculitis and renal involvement with subsequent elevated BP and cardiovascular events. Notably, in the present study, the time from HCV diagnosis to DAA treatment was significantly shorter in the subgroup with improved BP control thereafter, which may confirm that the longer duration of HCV infection determines the degree of irreversible vascular damage and, therefore, influences the net effect of HCV eradication on BP control. Recently, an increased augmentation index following viral eradication with DAA therapy was found in patients with advanced fibrosis (≥9.5 kPa) [25]. Of note, in patients with non-advanced fibrosis, the authors observed a stable augmentation index and significantly lower values of both office and central systolic pressure at the SVR12 time point [25]. In contrast, we observed a significant decrease of the normalized augmentation index in a subgroup with BP improvement, whereas any change in arterial stiffness, measured by pulse wave velocity, was found in our cohort, independently of BP changes after DAA treatment.

When considering the possible changes of endothelial function after successful HCV eradication with DAA, the reduction of endothelium-derived adhesion molecule levels at the SVR12 time point was confirmed by two small studies [26,27], but the improvement of FMD values was observed only in one group [26], and Davis et al. did not find changes in bedside microvascular reactivity, measured by peripheral arterial tonometry [27]. Of note, both studies were performed in individuals with normal renal function. In our cohort, we observed stable FMD values and significantly lower NMD values after DAA treatment; however, a repeated measurement with a double dose of nitroglycerin did not confirm the preceding finding. As we previously reported, the significant decrease of calcineurin inhibitor levels shortly after successful HCV eradication in this group [15], we may speculate that this effect was caused by an improved liver metabolic efficiency, which limited the biologic effect of a standard nitroglycerin dose used originally. Furthermore, the calcineurin inhibitor co-medication may also influence the endothelial function in our group [28], though the proportion of cyclosporine A- and tacrolimus-treated patients was similar in both study subgroups.

It is important to notice that both analyzed subgroups did not differ at baseline in terms of demographics, co-morbidity, or kidney graft function. Also, arterial stiffness and endothelial function measures, as well as liver function tests and liver stiffness measurements, were comparable. There were significantly higher baseline office systolic blood pressure and pulse pressure values, but not central blood pressure parameters, in subgroup 1. The posttreatment follow-up evaluation, along with the substantial BP control improvement, revealed a significant decrease of CAP values in these patients. Notably, in patients without known cause of chronic liver disease, increased CAP was shown to be a good indicator of fatty liver disease with metabolic abnormalities that manifest even before a sonographic fatty change appears [29]. 

In HCV-infected patients, an intrahepatic viral load directly enhances the liver steatosis [30], especially in the setting of multiple metabolic abnormalities (expansion of visceral adipose tissue, insulin resistance, reversible hypocholesterolemia, arterial hypertension, and hyperuricemia). On the other hand, in patients with non-alcoholic fatty liver disease (NAFLD), steatosis grade was the most important factor for endothelial dysfunction [31]. Furthermore, lower FMD values were shown in pediatric NAFLD patients, in whom systolic and diastolic blood pressures were significantly higher than in healthy controls, but also higher than obese children with normal livers [32]. Even more importantly, fatty liver was shown to be associated with 24-h systolic blood pressure and daytime diastolic blood pressure measurements [24]. Recently, Wang et al. demonstrated that NAFLD is independently associated with hypertension and blood pressure category [33]. In this study, the CAP value was the predictor of diastolic, but not systolic BP in stepwise analysis for the whole study group, not only in NAFLD participants [33]. Of note, CAP alone was not sufficient for predicting hypertension in this study. 

It is well known that in the population of KTRs, the measures of vascular damage are conditioned by many factors, including the duration of chronic kidney disease, pretransplant dialysis vintage, concomitant immunosuppressive regimen, and non-optimal kidney graft function. Thus, the possibility for marked improvement of vascular elasticity and distensibility after the successful HCV treatment is much lower than in the general population. Nonetheless, overall results of our investigation may indirectly confirm the notable role of decreasing liver steatosis in the BP control improvement in the study group.

Finally, FGF-21 levels are reported to increase in acute liver injury, but decrease in chronic hepatitis B patients [34], especially those with advanced fibrosis [35]. We analyzed FGF-21 concentrations before and after the successful treatment of chronic HCV infection. Its levels significantly increased only in patients with initially more advanced steatosis as shown by CAP and improved BP control after DAA therapy and HCV elimination. The concomitant decrease of liver steatosis after DAA therapy observed in our study is in line with previously published papers [36,37] and may play a significant role in improved BP control. 

The main limitation of our analyzed cohort was its small size, which limited multivariate analysis of factors independently associated with the BP control improvement. Therefore, our preliminary data should be investigated in a larger multicenter cohort. However, we included all eligible KTRs with the sustained elimination of chronic HCV infection. Of note, the structure of DAA regimen in our patients was uniform: All were treated with a sofosbuvir-based therapy. Another limitation was the method of OBP measurement used in this study, specifically the lack of unattended BP measurement, which eliminated the potential bias related to the ‘white coat’ effect. In addition, we did not perform the 24-h ambulatory blood pressure monitoring; however, we observed the central blood pressure values and found a strong association with OBP results. Lastly, we would like to acknowledge the lack of monitoring of the adherence to antihypertensive medication in our study.

## 5. Conclusions

In conclusion, we observed the clinically important improvement of both office blood pressure control and central aortic pressure parameters, including augmentation index, in half of our cohort of HCV-infected stable kidney transplant recipients after successful HCV eradication with a sofosbuvir-based DAA therapy. The concomitant decrease of liver steatosis, measured by the controlled attenuation parameter, was only observed in this subgroup of patients, which may suggest the potential underlying mechanism of beneficial hemodynamic changes. Further investigation would elucidate the pathomechanism of the observed improvement of blood pressure control, however, the increased availability of DAA therapy already resulted in pretransplant eradication of HCV infection in a majority of currently dialysis patients. We believe that patients receiving DAA therapy in the pretransplant period will benefit even more. Nevertheless, the confirmation of relatively moderate BP improvement in dialysis patients would be much more difficult due to procedural-related fluctuations in volemia. 

## Figures and Tables

**Table 1:** Characteristics of direct antiviral agents-treated kidney transplant recipients stratified to subgroups based on blood pressure control improvement in the follow-up period.

	OBP Control Improvement *n* = 14	No OBP Control Improvement *n* = 14	*p*
Age at the start of DAA therapy (years, means and 95%CI)	49.8 (42.8–56.8)	48.5 (42.2–54.9)	0.78
Sex (M/F)	8/6	11/3	0.23
Baseline BMI (kg/m^2^, means and 95%CI)	24.9 (22.8–26.9)	24.0 (22.3–25.7)	0.48
Follow-up BMI (kg/m^2^, means and 95%CI)	25.6 (23.5–27.6) ^##^	24.4 (22.6–26.3)	0.40
Baseline eGFR (mL/min/1.73 m^2^, means and 95%CI)	58.1 (45.1–71.2)	54.2 (36.3–72.1)	0.51
Follow-up eGFR (mL/min/1.73 m^2^, means and 95%CI)	54.6 (39.4–69.8)	51.7 (35.0–68.5)	0.51
Diabetes (*n* (%))	4 (28.6)	4 (28.6)	1.0
Duration of HCV infection (years, means and 95%CI)	11.0 (6.9–15.1)	16.4 (12.6–20.1)	<0.05
Dialysis vintage (months, median and IQR)	30 (18–59)	42 (20–83)	0.37
Time after KTx (months, means and 95%CI)	121 (56–186)	125 (80–169)	0.92
Calcineurin inhibitor (CyA/Tc) (*n*)	8/6	7/7	0.70
Baseline HCV viremia (IU/mL, median and IQR)	340,258 (29,882–1,149,502)	78,631 (14,847–330,000)	0.40

^##^*p* < 0.01 versus baseline. CI, confidence interval; IQR, interquartile range; OBP, attended office blood pressure; DAA, direct antiviral drug therapy; BMI, body mass index; eGFR, estimated glomerular filtration rate; KTx, kidney transplantation; CyA, cyclosporine A; Tc, tacrolimus.

**Table 2:** Office BP measurements and antihypertensive treatment before and after successful DAA therapy, divided into two subgroups based on changes in OBP control after treatment.

	OBP Control Improvement (*n* = 14)			*p*	No OBP Control Improvement (*n* = 14)			*p*
	Baseline	Follow-up	Δ		Baseline	Follow-up	Δ	
SBP (mmHg, mean and 95% CI)	145.5 (138.3–152.7)	125.1 (116.3–133.8)	−20.4 (−26.2–−14.6)	<0.001	135.6 (130.4–140.1)	130.4 (125.1–135.6)	−5.2 (−9.7–−0.8)	<0.05
DBP (mmHg, mean and 95% CI)	84.3 (78.8–89.8)	71.8 (67.0–76.6)	−12.5 (−16.5–−8.5)	<0.01	86.9 (83.6–90.1)	82.2 (79.3–85.1)	−4.6 (−9.6–0.3)	0.07
PP (mmHg, mean and 95% CI)	61.2 (52.7–69.7)	53.3 (43.2–63.3)	−7.9 (−14.1–−1.8)	<0.05	48.7 (43.8–53.6)	48.1 (42.1–54.2)	−0.6 (−5.3 – 4.2)	0.86
Number of antihypertensive drugs (mean and 95% CI)	2.5 (1.3–3.7)	1.9 (0.8–2.9)	−0.6 (−1.0- −0.3)	<0.05	1.9 (1.4–2.5)	2.2 (1.5–3.0)	0.3 (0.0–0.6)	<0.07

CI, confidence interval; OBP, attended office blood pressure; DAA, direct antiviral drug therapy; SBP, systolic blood pressure; DBP, diastolic blood pressure; PP, pulse pressure.

**Table 3:** The central blood pressure parameters and the measurements of arterial stiffness (pulse wave velocity (PWV)) and endothelial function (flow-mediated dilation, (FMD) and nitroglycerine-mediated dilation (NMD)) measured before and after successful DAA treatment in kidney transplant recipients in both study subgroups, based on changes in OBP control after treatment.

	OBP Control Improvement (*n* = 14)			*p*	No OBP Control Improvement (*n* = 14)			*p*
	Baseline	Follow-up	Δ		Baseline	Follow-up	Δ	
**Central blood pressure measurement**								
AoSys (mmHg, mean and 95%CI)	136 (124–148)	114 (104–124)	−21.8 (−30.1–−13.5)	<0.01	131 (121–141)	120 (114–127)	−10.6 (−19.4–−1.9)	0.06
AoDia (mmHg, mean and 95%CI)	89 (81–96)	77 71–83)	−11.6 (−19.2–−4.1)	<0.05	87 (81–92)	82 (76–87)	−5.0 (−12.8–−2.8)	0.19
AoPP (mmHg, mean and 95%CI)	47 (38–57)	37 (29–46)	−10.1 (−14.1–−6.2)	0.09	44 (37–51)	39 (33–44)	−5.6 (−12.3–1.1)	0.19
AIx@75 (%, median and IQR)	20.5 (8.6–25.1)	16.9 (2.0–20.2)	−4.4 (−13.4–−1.1)	<0.05	15.5 (10.6–21.1)	16.5 (8.2–21.8)	0.0 (−8.2–7.8)	0.85
**Arterial wall assessments**								
PWV (m/s, mean and 95%CI)	8.5 (7.4–9.7)	8.7 (7.5–9.8)	0.1 (−0.7–0.9)	0.65	8.3 (7.1–9.5)	8.0 (6.9–9.2)	−0.3 (−1.4–0.8)	0.55
FMD (%,median and IQR)	9.2 (8.1–12.5)	9.3 (4.9–17.9)	2.1 (−6.5–3.3)	0.88	9.9 (7.5–15.0)	9.6 (5.6–12.2)	−1.6 (−2.8–0.6)	0.36
NMD (%,mean and 95%CI)	12.9 (10.3–15.6)	11.1 (8.4–13.8)	−1.8 (−5.5–1.8)	0.30	11.8 (9.9–13.7)	6.3 (3.7–8.9)	−5.5 (−9.1–−2.0)	<0.001
NMD with double dose (%,mean and 95%CI)	12.9 (10.3–15.6)	13.4 (10.3–16.6)	0.5 (−2.2–3.2)	0.80	11.8 (9.9–13.7)	10.5 (5.7–15.3)	−1.3 (−6.0–3.5)	0.59

CI, confidence interval; IQR, interquartile range; OBP, office blood pressure; AoSys, aortic systolic pressure; AoDia, aortic diastolic pressure; AoPP, aortic pulse pressure; AIx75, aortic augmentation index normalized to heart rate of 75/min; PWV, pulse wave velocity; FMD, flow-mediated dilation; NMD, nitroglycerin-mediated dilation. For all comparisons of baseline values between two study subgroups, *p* values were >0.05.

## Supplementary Materials

The following are available online at https://www.mdpi.com/2077-0383/9/4/948/s1, Table S1: Laboratory parameters before and after successful DAA therapy, divided into subgroups based on changes in OBP control after treatment, Table S2: The results of liver function tests as well as the measurements of liver stiffness (LSM) and liver steatosis (controlled attenuation parameter—CAP) in kidney transplant recipients before and after the successful DAA therapy, divided into 2 subgroups based on the changes in OBP control after treatment.
