# Supplementary material for: Changes in Office Blood Pressure Control, Augmentation Index, and Liver Steatosis in Kidney Transplant Patients after Successful Hepatitis C Infection Treatment with Direct Antiviral Agents
Source: J Clin Med. 2020 Mar 30;9(4):948. doi: 10.3390/jcm9040948 (PMC7230312; doi:10.3390/jcm9040948)
Supplement: Supplementary file 1 [file jcm-09-00948-s001.pdf]

Supplementary Table S1. Laboratory parameters before and after successful DAA therapy, divided into subgroups based on changes in OBP control after treatment.

	**OBP control improvement (*N* = 14)**			***p***	**No OBP control improvement (*N* = 14)**			***p***
	**Baseline**	**Follow-up**	**Δ**		**Baseline**	**Follow-up**	**Δ**	
TC [mmol/l mean and 95% CI]	5.2 (4.8–5.6)	5.3 (4.8–5.9)	0.1 (−0.3–0.6)	0.74	4.7 (4.0–5.4)	5.1 (4.4–5.8)	0.4 (−0.2–1.1)	0.35
TG [mmol/l, mean and 95% CI]	1.5 (1.2–1.8)	1.4 (1.2–1.7)	−0.1 (−0.3–0.2)	0.63	1.6 (1.2–2.0)	1.5 (1.1–1.8)	−0.1 (−0.5–0.2)	0.61
Insulin [mU/l, median and IQR] *	9.0 (7.5–22.3)	10.8 (8.0–13.8)	−0.0 (−2.4–1.1)	0.35	11.5 (8.7–19.9)	12.1 (7.1–19.4)	−0.5 (−1.9–1.0)	0.55
HbA_{1c} [%,median and IQR]	5.4 (5.1–6.1)	5.7 (5.6–6.0)	0.2 (−0.4–0.4)	0.89	5.7 (5.3–6.9)	5.4 (5.1–6.5)	−0.4 (−0.8–0.3)	0.24
HOMA-IR [median and IQR]	2.4 (1.7–5.0)	2.7 (2.2–3.2)	0.1 (−1.4–0.4)	0.83	2.6 (1.9–4.7)	2.9 (1.4–4.8)	−0.3 (−0.5–0.4)	0.70

CI, confidence interval; IQR, interquartile range; TC, total cholesterol; TG, triglycerides; HbA_{1C}, glycated hemoglobin; HOMA-IR, insulin resistance as estimated by the homeostasis model assessment. For all comparisons of baseline values between two study subgroups, *p* values were >0.05.

Supplementary Table S2. The results of liver function tests as well as the measurements of liver stiffness (LSM) and liver steatosis (controlled attenuation parameter – CAP) in kidney transplant recipients before and after the successful DAA therapy, divided into 2 subgroups based on the changes in OBP control after treatment.

	**OBP control improvement (*N* = 14)**			***p***	**No OBP control improvement (*N* = 14)**			***p***
	**Baseline**	**Follow-up**	**Δ**		**Baseline**	**Follow-up**	**Δ**	
**Liver function tests**								
AST [IU/l, median and IQR]	41.5 (24.0–56.0)	17.0 (15.0–24.0)	−21.5 (−32.0–−9.0)	<0.01	33.0 (20.0–43.0)	19.0 (10.0–22.0)	−11.0 (−22.0–−9.0)	<0.01
ALT [IU/l, median and IQR] *	41.0 (26.0–80.0)	16.5 (10.0–20.0)	−28.5 (−39.0–−7.0)	<0.01	32.0 (22.0–51.0)	16.0 (10.0–23.0)	−16.0 (−21.0–−9.0)	<0.01
GGT [IU/l, median and IQR]	38.0 (19.0–70.0)	19.5 (14.0–27.0)	−16.5 (−26.0–1.0)	<0.05	37.5 (29.0–79.0)	19.5 (13.0–39.0)	−21.0 (−44.0–−14.0)	<0.01
TB [mg/dl, mean and 95% CI]	1.0 (0.8–1.2)	0.9 (0.7–1.1)	−0.1 (−0.3–0.1)	0.64	0.9 (0.7–1.0)	0.8 (0.6–0.9)	−0.1 (−0.3–0.1)	0.39
**Liver stiffness and steatosis assessments**								
LSM [kPa, median and IQR]	6.7 (5.4–9.6)	6.6 (5.8–9.2)	−0/5 (−0.6–1.2)	0.81	7.4 (6.1–11.5)	7.2 (5.6–9.0)	−0.9 (−2.3–0.9)	0.22
CAP [dB/m, mean and 95% CI]	241 (209–274)	209 (191–227)	−32 (−63–−1)	<0.05	221 (192–249)	216 (196–236)	−5 (−30–25)	0.63

CI, confidence interval; IQR, interquertile range; BP, office blood pressure; AST, aspartate aminotransferase; ALT, alanine aminotransferase; GGT, gammaglutamyltranspeptidase; TB, total bilirubin; LSM, liver stiffness measurement; CAP, controlled attenuation parameter. For all comparisons of baseline values between two study subgroups, *p* values were >0.05.
